# The Ubiquitous and Multifaceted Coenzyme Q

**DOI:** 10.3390/antiox13101261

**Published:** 2024-10-17

**Authors:** Luca Tiano, Plácido Navas

**Affiliations:** 1 Department of Life and Environmental Sciences (DISVA), Marche Polytechnic University, 60131 Ancona, Italy; 2 Centro Andaluz de Biología del Desarrollo, Universidad Pablo de Olavide-CSIC-JA, 41013 Sevilla, Spain; 3 CIBERER, Instituto de Salud Carlos III, 28029 Madrid, Spain

Coenzyme Q_10_ (CoQ_10_) is composed of a benzoquinone ring and an isoprenoid side chain attached to carbon 3 of the ring. The isoprenoid chain is species-specific, e.g., six isoprene units are present in *Saccharomyces cerevisiae*, CoQ_6_, and 10 isoprene units in humans, CoQ_10_. CoQ is present in all species and its basic structure is conserved. Until now, no natural or artificial molecule has been able to replace CoQ_10_ functions. CoQ_10_ has been proposed to be an essential component in respiratory-active respirasome and other supercomplexes of the electron transfer chain (ETC) [1]. Supercomplexes modulate the organization of the ETC according to the cellular metabolic requirements, contribute to respiratory efficiency, and limit reactive oxygen species (ROS) production partially through the prevention of ubiquinol accumulation in the respiratory chain [2,3].

The reduction of CoQ_10_ located in the ETC is also an obligatory step of different pathways essential for cellular homeostasis [4]. Recently, it has been shown that tumor growth depends on the ETC complex III oxidation of ubiquinol (CoQ_10_H_2_) [5]. To further understand the role of CoQ_10_ in cancer progression, it has been demonstrated that CoQ_10_ and UBIAD1, an enzyme involved in its biosynthesis, increase membrane fluidity, leading to increased cell stiffness, impair extracellular matrix-mediated oncogenic signaling, and reduce ferroptosis resistance in breast cancer, and UBIAD1 is needed to limit circulating tumor cell survival and lung metastasis formation [6]. It is important to consider that these complex growth mechanisms are based on the metabolic redox regulation pathways in which cells acquire and utilize electron carriers to maintain directional carbon flux, sustain reductive biosynthesis, and support antioxidant defense, as recently considered [7].

CoQ_10_ is also in plasma membranes and cholesterol particles in plasma, providing antioxidant protection [8]. Plasma membranes contain a trans-membrane redox system that maintains both vitamins E and C in its reduced state, via CoQ_10_ reduction, through the membrane-bound NADH-cytochrome *b*_5_ reductase 3 (CYB5R3) and NAD(P)H: quinone oxidoreductase 1 (NQO1). This system prevents ceramide-mediated apoptosis [9]. The overexpression of this system was observed to increase the life- and health-span of mice, modulating lipid metabolism and improving respiration [10].

Additionally, ferroptosis is prevented by the ferroptosis suppressor protein 1 (FSP1)/apoptosis-inducing factor mitochondria-associated 2 (AIFM2) reduction of CoQ_10_ in plasma membranes, acting in parallel with glutathione-dependent lipid hydroperoxidase glutathione peroxidase 4 (GPX4) [11,12].

In eukaryotes, the CoQ_10_ biosynthesis pathway is carried out by proteins encoded by nuclear *COQ* genes that interact, generating a CoQ_10_ biosynthesis complex known as CoQ synthome [13]. However, not all enzyme reactions and the regulation of CoQ synthome have been clarified [14]. This year, the function of COQ4 in the decarboxylation and hydroxylation reactions of carbon in position 1 was demonstrated [15]. New advances in CoQ biosynthesis introduced the recently discovered protein RTN4IP1/OPA10 as a mitochondrial component required in this pathway, increasing its complexity and regulation [16]. New advances in CoQ biosynthesis research have provided insights into the molecular mechanisms of this complex pathway through the in vitro construction of the animal CoQ biosynthesis metabolon [17] and the reconstruction of *E. coli Ubi* metabolon [18].

Defects in CoQ synthome components or its regulatory elements lead to primary CoQ_10_ deficiencies, inducing highly heterogeneous clinical phenotypes and thus creating difficulties in establishing genotype–phenotype correlations; these deficiencies manifest as defects in the skeletal muscle, central and peripheral nervous systems, kidney, and heart. These are rare autosomal recessive conditions characterized by reduced levels of CoQ_10_ in tissues and organs [19].

All these topics were presented and discussed at the 10th Conference of the International Coenzyme Q_10_ Association that took place from 12 to 15 May 2022 in Hamburg, Germany. For the first time, the conference was hosted in collaboration with an industrial partner, Beiersdorf, highlighting the growing importance of applied research in this field. This partnership underscored the significance of bridging the gap between academic research and real-world industrial applications, and it provided a fertile ground for the research presented here.

As we draw this Special Issue of *Antioxidants* to a close (https://www.mdpi.com/journal/antioxidants/special_issues/Antioxidants_Coenzyme) (accessed 17 October 2024), we reflect on the outstanding research contributions presented and discussed at this conference, highlighting the multifaceted roles of CoQ_10_. This Special Issue has garnered six significant contributions that delve into the multifaceted roles of CoQ_10_, emphasizing its biological importance, therapeutic potential, and the challenges associated with its deficiency. Across these studies, CoQ_10_ emerges as a vital molecule, not only for its well-known antioxidant properties but also for its crucial role in cellular energy production, bioenergetics, and metabolic regulation.

One key focus of this Special Issue, encapsulated by one study, is the cellular uptake and distribution of CoQ_10_, demonstrated through innovative techniques like X-ray fluorescence (XRF) imaging. This study provides valuable insights into how CoQ_10_ is absorbed by skin cells, laying the groundwork for its potential use in anti-aging and oxidative stress protection therapies. Another study explores the role of CoQ_10_ in brown adipose tissue (BAT), highlighting its regulatory effect on thermogenesis through the expression of uncoupling protein 1 (UCP1). The findings suggest that CoQ_10_ deficiencies can impair energy metabolism and contribute to metabolic disorders such as obesity, pointing to its therapeutic relevance in these conditions. CoQ deficiency in BAT results in the activation of the integrated stress response with a suppression of UCP1 but an induction of FGF21 expression. A BAT-specific CoQ deficiency interacts with inguinal white adipose tissue (iWAT), causing an increase in the browning of iWAT in deficient animals [20].

In this context, long-term CoQ_10_ supplementation lowered lipid peroxidation particles, conferring health benefits by decreasing cardiovascular mortality and improving the functional classification class of the New York Heart Association (NYHA) [21,22]. Selenium associated with CoQ_10_ supplementation also activated selenoprotein P, providing benefits to inflammation, the length of telomeres, quality of life, and mortality [23]. This is partially explained by the regulation of vascular flexibility due to the mitigation of endothelial inflammatory activation caused by CYB5R3, assisting in NOX4-dependent H_2_O_2_ generation via CoQ_10_ [24].

As indicated above, CoQ_10_ supplementation was proposed as a therapy to improve the health of people suffering from various disorders such as inflammation, metabolic syndrome, and male infertility, among others [25]. A trial focused on CoQ_10_ supplementation demonstrated an improvement of liver steatosis and endothelial, vascular and myocardial function in patients with metabolic dysfunction-associated steatotic liver disease [26].

Furthermore, a comprehensive review of CoQ_10_ biosynthesis and deficiencies underscores the diagnostic challenges and therapeutic gaps in addressing both primary and secondary CoQ_10_ deficiencies. Secondary CoQ_10_ deficiencies are particularly challenging because they are caused by mutations in genes that are not involved in CoQ synthome or its regulation. These diseases do not respond to CoQ_10_ supplementation, meaning that new personalized therapies should be developed [27]. The need for improved supplementation strategies and better drug delivery methods is highlighted, particularly for crossing the blood–brain barrier. Complementing this, an AlphaFold structural analysis of the COQ2 enzyme, central to CoQ_10_ biosynthesis, sheds light on how mutations disrupt CoQ_10_ production, leading to primary CoQ_10_ deficiencies. Together, these studies offer a cohesive and detailed exploration of CoQ_10_, reinforcing its critical role in health and advancing the conversation on how to better harness its therapeutic potential. According to these contributions, it should be emphasized that as CoQ_10_ supplementation is the treatment for primary CoQ_10_ deficiencies, its bioavailability should be the focus of current and future translational research, including recent attempts made to develop new formulations and use CoQ synthome intermediates for personalized therapies [28,29,30].

To further understand the lack of positive response to CoQ_10_ supplementation in secondary CoQ_10_ deficiencies, it is important to consider that most patients suffering from these diseases show symptoms in the perinatal stage, which indicates that the pathomechanisms are established during their development [31]. Pertaining to this, prenatal treatment with CoQ_10_ in a mouse model of secondary CoQ deficiency demonstrated a higher benefit than the treatment of the adult animal, indicating that providing CoQ_10_ supplementation to pregnant mothers should be recommended [32].

This Special Issue also addresses the broader role of mitochondrial reactive oxygen species (mtROS) in the aging population, illustrating how disruptions in redox signaling can lead to age-related diseases. This review suggests a shift from traditional antioxidant approaches to therapies that restore balanced redox signaling. In the same vein, the enhanced bioavailability of CoQ_10_ through phytosome-based formulations like ubiqsome is explored, offering promising advancements in CoQ_10_ delivery, especially in muscle cells, where its uptake is typically limited.

Looking ahead, the 11th Conference of the International CoQ_10_ Association will be hosted by the University of Copenhagen from 16 to 19 June 2025 (https://icq10a.com/). This upcoming event promises to build on the strong foundation laid by previous conferences, offering new opportunities to engage with cutting-edge research on CoQ_10_. We are pleased to announce that this year’s conference will include, for the first time, an educational day on June 16. This pre-conference event is designed to bring CoQ_10_ science to a younger audience, with activities tailored for college undergraduates and recent graduates in biomedical disciplines or medicine. It will offer an informative introduction to CoQ_10_ research while encouraging curiosity and providing guidance on potential career paths in this rapidly evolving field.

Key topics of the upcoming conference will include new advances in CoQ_10_ biosynthesis, the use of precursors to increase CoQ_10_ production, primary and secondary CoQ_10_ deficiencies, and clinical applications in areas such as cognitive health, persistent COVID, cardiovascular disease, and heart failure. In addition, sessions will cover the bioavailability of CoQ and its role in sport, physical activity, and healthy aging.

We hope this Special Issue serves as both a reference point and an inspiration for ongoing and future research into CoQ_10_. We extend our heartfelt thanks to all contributing authors for their exceptional work and to the reviewers for their invaluable feedback. We would also like to express our gratitude to the editorial team of *Antioxidants* for their support throughout this process. Together, we have curated a collection of papers that not only advances the science of CoQ_10_ but also stimulates the development of new research perspectives and future collaborations.

We invite all our readers and contributors to join us in Copenhagen, June 2025, where we will continue to explore and expand the boundaries of CoQ_10_ research.
